# Does stroke location predict walk speed response to gait rehabilitation?

**DOI:** 10.1002/hbm.23059

**Published:** 2015-11-19

**Authors:** P. Simon Jones, Valerie M. Pomeroy, Jasmine Wang, Gottfried Schlaug, S. Tulasi Marrapu, Sharon Geva, Philip J. Rowe, Elizabeth Chandler, Andrew Kerr, Jean‐Claude Baron, for the SWIFT‐Cast investigators

**Affiliations:** ^1^ Stroke Research Group, Department of Clinical Neurosciences University of Cambridge Cambridge United Kingdom; ^2^ Acquired Brain Injury Rehabilitation Alliance, School of Health Sciences University of East Anglia Norwich United Kingdom; ^3^ Department of Neurology Beth Israel Deaconess Medical Center, Harvard Medical School Boston Massachusetts; ^4^ Bioengineering Unit University of Strathclyde Glasgow United Kingdom; ^5^ Centre Hospitalier Sainte‐Anne Inserm U894, Sorbonne Paris Cité Paris France

**Keywords:** MRI, voxel‐based lesion–symptom mapping, cortico‐spinal tract, ambulation, recovery

## Abstract

**Objectives:**

Recovery of independent ambulation after stroke is a major goal. However, which rehabilitation regimen best benefits each individual is unknown and decisions are currently made on a subjective basis. Predictors of response to specific therapies would guide the type of therapy most appropriate for each patient. Although lesion topography is a strong predictor of upper limb response, walking involves more distributed functions. Earlier studies that assessed the cortico‐spinal tract (CST) were negative, suggesting other structures may be important.

*Experimental Design*: The relationship between lesion topography and response of walking speed to standard rehabilitation was assessed in 50 adult‐onset patients using both volumetric measurement of CST lesion load and voxel‐based lesion–symptom mapping (VLSM) to assess non‐CST structures. Two functional mobility scales, the functional ambulation category (FAC) and the modified rivermead mobility index (MRMI) were also administered. Performance measures were obtained both at entry into the study (3–42 days post‐stroke) and at the end of a 6‐week course of therapy. Baseline score, age, time since stroke onset and white matter hyperintensities score were included as nuisance covariates in regression models.

*Principal Observations*: CST damage independently predicted response to therapy for FAC and MRMI, but not for walk speed. However, using VLSM the latter was predicted by damage to the putamen, insula, external capsule and neighbouring white matter.

**Conclusions:**

Walk speed response to rehabilitation was affected by damage involving the putamen and neighbouring structures but not the CST, while the latter had modest but significant impact on everyday functions of general mobility and gait. *Hum Brain Mapp 37:689–703, 2016*. © **2015 Wiley Periodicals, Inc.**

## INTRODUCTION

Around a third of stroke survivors are unable to ambulate 6 months after stroke [Alexander et al., 2009], contributing a large portion of functional impairment and lost independence. Accordingly, rehabilitation aimed at recovering independent ambulation is an important part of post‐stroke therapy, using various techniques that include apparatus‐supported therapy such as treadmill exercise, balance activities and orthoses. However, the type of therapy that would best benefit each individual patient remains uncertain, and currently post‐stroke therapy decisions are made on a subjective basis. Therefore, predictors of response to therapy, that is, the gain in functional scores between baseline and final assessments, would be of considerable value in the clinical setting as they would point to the type and amount of therapy most effective in each individual. This would in turn maximize the effects of therapy and enhance recovery for each particular lesion type.

Although previous studies have disagreed regarding the role of some clinical variables such as age, lesion volume and white matter small vessel lesion load as predictors of response to standard therapy [Burke et al., 2014; Cramer et al., 2007; Dawes et al., 2008; Dobkin et al., 2014; Held et al., 2012; Jorgensen et al., 1995; Kollen et al., 2005; Lam et al., 2010; Lindenberg et al., 2012; O'Shea et al., 2014; Stinear et al., 2007], time since stroke onset has been consistently found to influence, albeit weakly, response to rehabilitation therapy [Kollen et al., 2005; Lam et al., 2010; O'Shea et al., 2014; Stinear et al., 2007]. Another, probably stronger clinical predictor is baseline impairment [Burke et al., 2014; Cramer et al., 2007; Dawes et al., 2008; Dobkin et al., 2014; Kollen et al., 2005; Lindenberg et al., 2012; O'Shea et al., 2014; Riley et al., 2011; Stinear et al., 2007]. However, lesion topography is generally considered the strongest potential predictor of response to therapy after stroke.

Damage to the cortico‐spinal tract (CST), and particularly to the portion of the CST originating from the primary motor cortex (M1), has consistently been reported as a major determinant of *final outcome*, including global impairment [Pineiro et al., 2000; Puig et al., 2010, 2011, 2013] and particularly upper limb weakness [Feng et al., 2015; Kim et al., 2013; Lindenberg et al., 2010; Lo et al., 2010; Maraka et al., 2014; Qiu et al., 2011; Rosso et al., 2013; Schaechter et al., 2009; Schulz et al., 2012; Stinear et al., 2007; Zhu et al., 2010]. However, information regarding how lesion topography affects walking outcome is scarce. As expected, based on established CST neuroanatomy, in three studies, leg weakness was significantly related to CST damage measured as involvement of the posterior limb of the internal capsule (PLIC) [Jayaram et al., 2012; Lee et al., 2005] or corona radiata [Alexander et al., 2009], or overlap of the lesion with the whole extent of the CST [Jayaram et al., 2012]. However, walking and gait entail considerably more complex functions than just will‐guided leg strength, and consequently are expected to involve more extensive systems than solely the CST [Perennou and Hillier, 2014]. Accordingly, neither Jayaram et al. [2012] nor Dawes et al. [2008] found a significant relationship between lesion CST overlap and walking speed in the chronic stage post‐stroke. In one study, the amount of CST damage predicted ambulation outcome assessed with the functional ambulation category (FAC) scale [Kim et al., 2013], possibly suggesting a differential role of CST damage on walking speed versus actual ambulation. Interestingly, Alexander et al. [2009] found that damage to the putamen, insula and external capsule was related to gait asymmetry, while abnormal activation of the basal ganglia, insula, secondary somatosensory area, or supplementary motor and premotor cortex during leg movement have also been found associated with impaired lower limb movement [Dobkin et al., 2004; Enzinger et al., 2009; Mihara et al., 2012; Miyai et al., 2003]. Overall, therefore, other structures beyond the CST may be involved in walking impairment after stroke.

Although as just described, there is relatively abundant knowledge regarding the lesion anatomy of post‐stroke motor impairment, particularly for the upper limb, much less is known of the predictive value of lesion topography for *response to therapy*, that is, the change in clinical measures of motor deficit following participation in characterized rehabilitation intervention. Several studies have consistently reported that CST damage predicts response of upper limb motor deficit to therapy [Lindenberg et al., 2012; Nouri and Cramer, 2011; Riley et al., 2011; Stinear et al., 2007]. In those studies, however, a substantial fraction of the variance in response to therapy remained unexplained, suggesting other systems are also involved. So far, two studies only have addressed the predictive value of CST damage for walking recovery. Both showed no significant relationship of CST lesion overlap [Burke et al., 2014; Dawes et al., 2008], further suggesting that CST is not a strong determinant of recovery of walking speed and that other structures are probably involved.

In the present prospective study on a substantial sample of stroke survivors, we used volumetric CST lesion load measurement [Zhu et al., 2010] to assess the relationship between CST damage and response of walking speed to ambulation rehabilitation. In addition to specific CST damage volumetry, we also used voxel‐based lesion–symptom mapping (VLSM) [Bates et al., 2003] to assess the role of non‐CST structures. Finally, in addition to walk speed, two clinical scales measuring everyday mobility were also obtained.

## SUBJECTS AND METHODS

### Patients

Participants were prospectively recruited in the Soft‐Scotch Walking Initial FooT (SWIFT) Cast randomized controlled trial. The trial evaluated the efficacy of augmenting conventional therapy, which could include standard ankle–foot orthoses, with a specific ankle–foot cast (SWIFT Cast) to enhance walking recovery [Pomeroy et al., 2012]. Only those patients who had agreed to undertake magnetic resonance imaging (MRI), which was optional per protocol, were eligible for this study. As the trial was completely neutral [Pomeroy et al., 2015], both patient groups were merged for the analysis, as done in two previous publications [Burke et al., 2014; Cramer et al., 2007].

As detailed elsewhere [Pomeroy et al., 2012], inclusion criteria were: aged more than 18 years; 3–42 days after stroke; infarct or haemorrhage; subjects in whom gait rehabilitation was judged both necessary and potentially useful, namely presence of gait abnormalities (knee hyperextension and/or abnormal initial floor contact) but able to take at least three steps while supported by two people; no contractures at hip, knee, ankle, or forefoot or loss of skin integrity over the paretic foot/lower limb; able to follow a 1‐stage command, that is, sufficient communication/orientation for interventions in this trial; and otherwise, physically fit for rehabilitation. Potentially eligible patients were enrolled into the study as soon as they were able to take at least three steps while supported by two people. Nature of the stroke (i.e., ischemic or haemorrhagic) and ischemic stroke sub‐type (i.e., large‐vessel or lacunar) and topography were not part of the inclusion criteria as the aim of this study was to recruit a sample as representative of patients referred for gait therapy to a rehabilitation unit as possible.

The protocol was approved by the relevant Regional Ethics Committees and registered on a clinical trials database (ISRCTN 39201286). Each participant gave signed informed consent.

### Clinical Assessment

Functional performance measures were taken at entry into the study and the end of 6 weeks of intervention phase [Pomeroy et al., 2012]. The primary outcome measure was average walking speed (m/s). Walking speed was measured using a 2‐D light switch and video system which has good reliability [Ugbolue et al., 2013]. Walking speed was chosen as the primary measure for the investigation of clinical efficacy as it (a) has international clinical utility; (b) was the target functional improvement for a SWIFT Cast; (c) is a meaningful functional outcome for stroke survivors; and (d) is used widely in stroke rehabilitation trials.

In addition to walk speed, which is an objective metric for walking ability, two functional mobility scales that incorporate other factors than just motricity were obtained as secondary outcomes, namely the FAC and the modified rivermead mobility index (MRMI) [Lennon and Johnson, 2000]. The FAC scale has six levels [Holden et al., 1984] ranging from unable to walk (score 0) to able to walk independently (score 5), and includes components of balance and supporting use of the upper limbs for the scores up to 4. This measure has been found to have strong inter‐rater and test–retest reliability [Mehrholz et al., 2007]. The MRMI measures functional mobility across eight tasks including turning over in bed, sitting up from the lying position, sitting balance, transferring to a chair, sitting to standing, walking indoors and ascending stairs [Walsh et al., 2010]. Each MRMI task ranges from “unable to perform” (score 0) to “independent” (score 5). The amount and content of the physical therapy received by participants is described elsewhere [Pomeroy et al., 2016]. The mean number of trial‐specific rehabilitation sessions per participant was nine, with each session lasting a mean of 40 (SD 16) min over the 6‐week intervention phase.

Given the aim of this study to assess the anatomical predictors of response to therapy, the difference between the baseline and outcome measures for the three behavioural variables detailed above were calculated and used in all statistical analyses below, unless indicated otherwise.

### MRI Data Acquisition

The imaging sub‐study was part of the prospective trial design [Pomeroy et al., 2012], and aimed to address the question Does stroke location predict response to gait rehabilitation?, “predict” being used here in the statistical perspective, not at the individual subject level, that is, is there a location that correlates with response to therapy across the group? Patients who agreed to undergo scanning underwent structural MRI including a whole‐brain “volume” MPRAGE T1‐weighted sequence and a T2‐weighted FLAIR sequence (see below). To have an accurate delineation of the cerebral lesion, this session was undertaken 3–8 weeks after stroke onset so that the lesion had stabilized [Gaudinski et al., 2008], that is, without remaining swelling from oedema but before substantial shrinkage develops [Deoni et al., 2008; Gale and Pearson, 2012].

Scanning was performed at two recruiting centres using similar Siemens 1.5T scanners (Avanto and Magnetom Sonata, respectively). Whole‐brain T1‐weighted MRI scans were acquired using a standard magnetization‐prepared rapid acquisition gradient‐echo (MPRAGE) sequence, which was followed by a standard whole‐brain T2‐weighted fluid‐attenuated inversion recovery (FLAIR) sequence with 4‐mm thick slices and 1‐mm interslice gap.

### Lesion Delineation

Using MRIcron (http://www.cabiatl.com/mricro/index.html), the stroke lesion was delineated on FLAIR images (with help from the T1‐MPRAGE images whenever appropriate) by a stroke neurologist with imaging experience (J‐CB), blinded to all clinical data except the side of the stroke. In addition, white matter hyperintense lesions on FLAIR were rated according to the standard Fazekas scale, from 0 (absent) to 3 (maximum) [Fazekas, 1989].

### Image Processing

All image processing was performed in SPM8 (Wellcome Trust Centre for Neuroimaging, http://www.fil.ion.ucl.ac.uk/spm/software/spm8). The FLAIR images were coregistered to the T1 images and the T1 images were resliced to the FLAIR space. The lesions were smoothed using the SPM masking option of MRIcron [Rorden et al., 2007] (http://www.cabiatl.com/mricro/mricro/mricro.html). T1 images were transformed into Montreal Neurological Institute (MNI) space using the unified Segmentation and Warping process with lesion cost function masking [Andersen et al., 2010; Brett et al., 2001] and the transformation parameters were applied to the original lesions using nearest‐neighbour interpolation to place the lesions in standard space.

For the analyses described below, all right‐sided lesions were flipped onto the left hemisphere to permit comparison across the whole group.

### CST Lesion Load

The aim of this analysis was to assess the relationship between the amount of damage to the CST and the clinical measures across the patient sample. For each subject, the probabilistic volume overlap of their lesion with the CST was computed according to the weighted‐CST lesion load (wCST‐LL) method [Zhu et al., 2010]. The wCST‐LL was calculated by weighing each slice of overlap with the CST by the ratio of the maximum cross‐sectional area of the CST over the cross‐sectional area of that specific slice. This weighing option corrects for the narrowing of the CST descending into the PLIC from the motor cortex. In contrast to Zhu et al [2010], the canonical CST tract used in this study was determined by a probabilistic fibre tracking approach using FSL 3.1.2 (http://www.fmrib.ox.ac.uk) and DTI data from 12 healthy elderly control subjects (9 male; mean age: 56.5 ± 14.8 years) [Feng et al., 2015]. Pre‐processing steps included correction for eddy current effects, skull stripping as well as estimation and fitting of diffusion parameters. Single slice regions of interest (ROIs) were drawn on the FA images in the pons, PLIC and the white matter underlying the posterior part of the precentral gyrus. Exclusion ROIs were drawn on the superior and medial cerebellar peduncle to exclude fibres to the cerebellum, as well as the middle sagittal region covering the brain stem and corpus callosum to exclude trans‐hemispheric fibres. Probtrackx (http://www.fmrib.ox.ac.uk/fsl/fdt/fdt_probtrackx.html) was used to track fibres from the pons ROI as the seeding region. Tracts were normalized to the SPM5 T2 template implemented in MATLAB (The Mathworks, Natick, MA), which was achieved by normalizing the DWI image to the SPM 5 T2 template, and then applying the normalization parameter to each CST tract. A 50th fractional anisotropy percentile threshold was applied to each CST fibre, and then the 12 tracts were each binarized and summed to create the canonical CST. wCST‐LL values obtained using this canonical CST significantly (*P* < 0.0001) predicted 3‐month Fugl‐Meyer (FM) [Fugl‐Meyer et al., 1975] upper extremity sensorimotor outcome in an unrelated dataset of 76 subjects [Feng et al., 2015]. Supporting Information, Figure 1 illustrates the excellent coverage of motor fibres originating from the M1 leg area.

**Figure 1:**
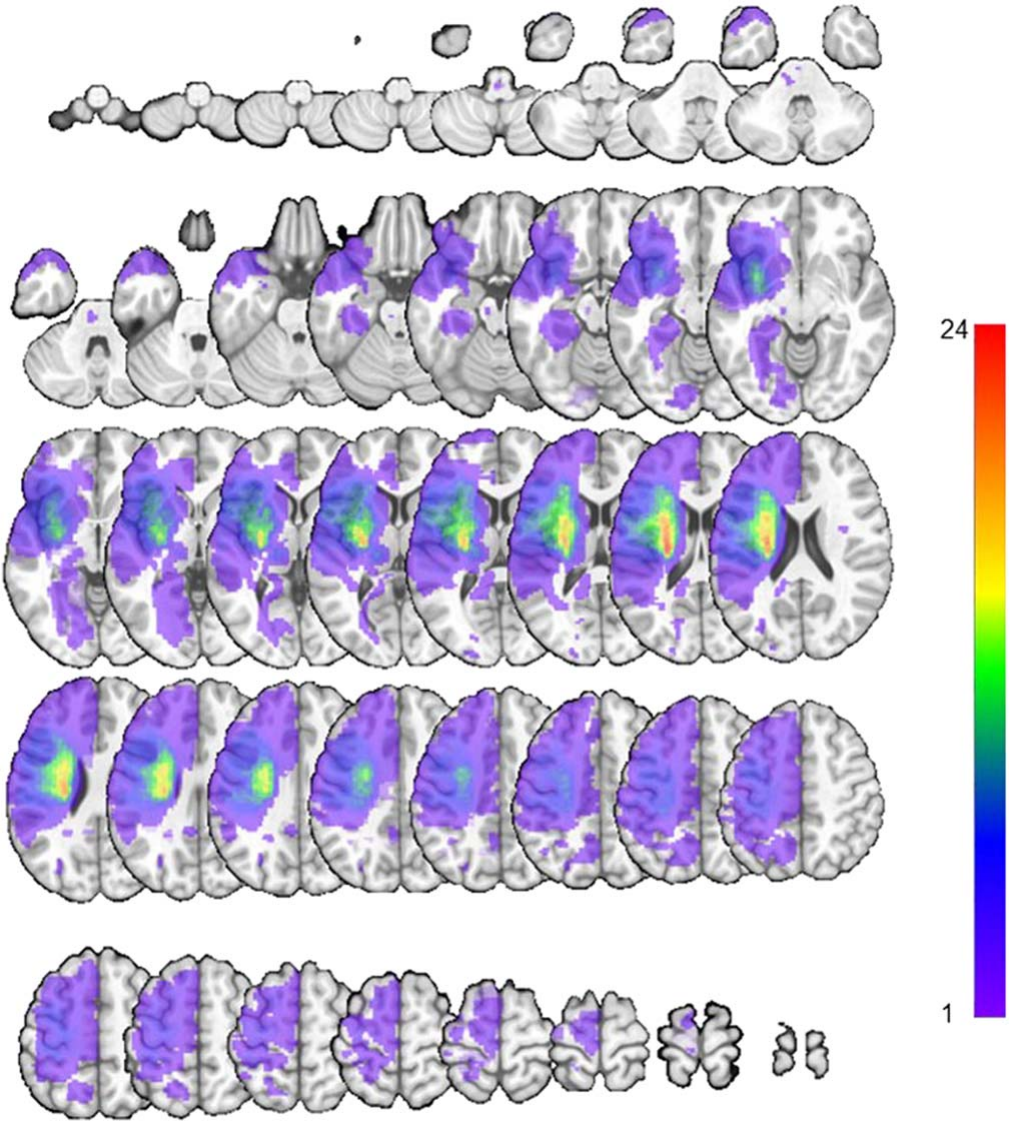
Lesion overlap map from the 50 participants overlaid on a standard MNI space brain after the right‐sided lesions had been flipped to the left side (see Methods section), and projected onto the whole set of axial slices from the canonical normal subject T1‐weighted MRI in Montreal Neurological Institute (MNI) space. The number of participants in each pixel is shown on the pseudo‐colour scale on the right. The maximum number of participants with a lesion for any voxel was 24 (red colour) and involved the striato‐capsular area and corona radiata. [Color figure can be viewed in the online issue, which is available at http://wileyonlinelibrary.com.]

### VLSM

VLSM was performed using vlsm2 version 2.53 [Bates et al., 2003] (http://www.neuroling.arizona.edu/resources.html). Each VLSM analysis identified clusters of voxels with statistically significant *t*‐values comparing voxelwise subjects’ clinical measures with lesions to those without lesions, and identified a peak *t*‐value within each significant cluster. Note that to avoid spurious results due to low numbers of lesioned voxels, only voxels lesioned in at least six participants were tested. The VLSM analysis involved first creating a *t*‐value map showing voxels with greatest difference in responses to lesioned and unlesioned status, thresholded at an uncorrected default cut‐off of *P* < 0.005. To correct for multiple comparisons the significance of the cluster was then assessed by randomly permuting the measures 5000 times between subjects, that is, nonparametrically. Only clusters with a peak *t*‐values in the top 5% of those generated randomly were considered significant (*P* < 0.05, permutation corrected).

To determine as objectively as possible the anatomical structures involved by the significant clusters, the location of each cluster was labelled according to the Hammers atlas [Hammers et al., 2003; Heckemann et al., 2006] (http://www.brain-development.org) for grey matter structures, the John Hopkins University (JHU) white matter tracts atlas [Hua et al., 2008], and where possible the Anatomy [Eickhoff et al., 2005] atlas for Brodmann's areas (BAs). We used the JHU tract atlas to assess the overlap of VLSM clusters with white matter tracts, including the CST. For each label, the percentage of the cluster overlapping with the given structure/tract/BA was obtained and tabulated.

### Statistical Analysis

#### Clinical data

Descriptive statistics were used to present the clinical data and their inter‐relationships. Continuous measures were summarized by mean and standard deviation or 95% confidence interval, and categorical data by median with interquartile range. Correlations were made using Kendall's Tau; corrections for multiple tests were deemed unnecessary given the descriptive aim. Statistical analyses were performed in SPSS 21 (IBM SPSS Statistics for Windows, Version 21.0. Armonk, NY: IBM Corp). Because a relatively large sample was used in this study, interpretation of the findings from correlations was not based only on the *P* value, which indicates the presence of a statistically significant relationship, but also on the *r* value, which assesses the strength of the relationship. In this study, we considered tau ≥ 0.6 to represent a strong relationship.

#### wCST‐LL analysis

The analysis of the relationship between wCST_LL and response to therapy for each of the three clinical scales was carried out with age, Fazekas score, time from stroke onset to baseline performance measures (to be referred to as “Time” below), and baseline score for the considered variable as added nuisance covariates. Lesion volume was not included as covariate as this can cause spurious results given the relationship between stroke size and topography according to vascular territories [Nachev, 2014]. In addition, there was not even a trend of a correlation between lesion volume and response to therapy for any of the three clinical variables assessed (*r* range: 0.01–0.06, all *P* > 0.53; data not shown). Multiple regressions were carried out for Walk speed and MRMI which are continuous and multiple categories variables, respectively, while for FAC, which has only five categories, ordinal regression was carried out.

#### VLSM

As with wCST‐LL, all four covariates described above were added in the VLSM analysis for each of the three variables.

## RESULTS

### Patient Characteristics and Behavioural Scores

Of the 105 trial participants, 56 consented to MRI but four were not suitable for inclusion in the image analysis (one had a hemi‐craniectomy and another had marked hydrocephalus entailing marked brain distortion, one declined study approval after the MR session, and one had no visible lesion on MR), leaving 52 subjects with adequate MRI for this study. Due to practical difficulties in obtaining MRI slots for this research, some scans were carried out slightly later than expected (mean time of MR relative to stroke onset: 52 days; range 17–74 days), even sometimes a few days after end of therapy. Because outcome clinical scores were not available in two additional patients, the final analysis was on 50 subjects.

Table 1 shows the patient demographics. The median time from stroke to enrolment in the trial was 16 days (range: 3–42 days). This subset of 50 subjects did not significantly differ from the remaining 53 trial subjects in any demographic or baseline clinical measure (data not shown). This material was made of 41 ischemic and 9 haemorrhagic strokes, of which four were hemispheric, four supratentorial deep‐seated and one involved the brain‐stem. Of the ischemic strokes, 22 were MCA‐territory strokes (eight of which were purely deep‐seated and two associated with posterior cerebral artery infarction), two were anterior cerebral artery (ACA) strokes, one was an anterior choroidal artery stroke, and 16 were lacunar infarcts (three of which located in the brainstem).

**Table 1:** Baseline characteristics of the subjects (*N* = 50) showing median (interquartile range) and range unless otherwise stated

Male/female	28/22
Left/right	25/25
Infarct/haemorrhage	41/9
Age (years)	64.6 (15.0)*a*, 27–100
Time to baseline assessment (days)	16.0 (9–25), 3–42
Fazekas score	2 (1–2), 0–3
Lesion volume (cm^3^)	4.4 (0.8–28.1), 0.05–188.18

Mean (SD).

Table 2 shows the functional scores of the participants at baseline and 6‐week outcome, and the change from baseline to outcome. There was significantly improved performance following treatment in all three functional measures.

**Table 2:** Summary functional performance measure (median and interquartile range unless otherwise stated; *N* = 50)

	Baseline	Outcome	Change	Effect size	*P* value^a^
Walk speed (m/s)	0.00 (0.00–0.25)	0.49 (0.18–0.71)	0.24 (0.00–0.51)	0.69	<0.000
MRMI	24 (19–32)	37 (34–38)	10 (4–15)	0.73	<0.000
FAC	1 (0–2)	4 (4–4)	3 (1–4)	0.58	<0.000

FAC = Functional Ambulation Category; MRMI = Modified Rivermead Mobility Index.

Change means the difference between Outcome and Baseline, that is, response to therapy. Effect sizes are from Cohen's r^2^ = Wilcoxon $\frac{Z}{\surd N}$. Small effect size (0.01–0.06); medium effect size (0.06–0.14); large effect size (>0.14).

a Wilcoxon signed rank test.

There were significant positive correlations regarding change in scores among all three clinical measures (all *P* < 0.03), particularly between FAC and MRMI (*P* < 0.001). However, they were weak between Walk speed and the other two scales (highest tau value: 0.386), while the tau value between MRMI and FAC was 0.611, indicating that a large part of the variance remained unexplained. Accordingly, the wCST‐LL and VLSM analyses were conducted for each variable separately, which also was justified by the marked differences in the everyday functions they assess (see Discussion section).

Table 3 shows the correlations of response to therapy for each behavioural measure with age, Fazekas score, time and baseline score. Age significantly but weakly negatively affected FAC score change. Baseline score was a strong predictor of response of FAC and MRMI scores, but not for Walk speed. The negative correlations indicated that the worse the initial score, the larger the absolute behavioural gain from therapy. Finally, Time significantly predicted score change for all three variables, again in the expected negative direction, but the correlations were weak.

**Table 3:** Correlation of response to therapy for the three behavioural measures with four baseline variables (Kendall's Tau)

	Age (years)	Fazekas score	Baseline score	Time from stroke to baseline
Walk speed	−0.19 (*P* = 0.06)	−0.18 (*P* = 0.10)	−0.21 (*P* = 0.07)	−0.23 (*P* < 0.03)
FAC	−0.25 (*P* < 0.02)	−0.04 (*P* = 0.76)	−0.60 (*P* < 0.001)	−0.26 (*P* < 0.02)
MRMI	−0.11 (*P* = 0.29)	0.01 (*P* = 0.91)	−0.65 (*P* < 0.001)	−0.26 (*P* < 0.01)

### wCST Lesion Load Analysis

One of the lesions was in the medulla and outside the standardised control CST, so this analysis was run on 49 subjects. Tables 4, 5, 6 show the results of the multiple regressions testing the correlation between the three response‐to‐therapy variables and wCST‐LL adjusting for age, Fazekas score, time and baseline score. Results from the multiple regression analysis revealed that CST damage did not significantly predict Walk speed change (*P* = 0.60), but significantly impacted changes in both FAC and MRMI (*P* = 0.030 and 0.024, respectively), albeit not strongly so, all in the negative, that is, biologically expected direction. Baseline score had a strong influence on FAC and MRMI, but only a weak—albeit significant—influence on Walk speed. Time modestly but significantly influenced FAC and MRMI. Age significantly influenced FAC response only. Interestingly, Walk speed was the least well‐predicted variable by the five covariates considered together.

**Table 4:** Multiple regressions to predict Walk speed response to therapy (*N* = 49 subjects)[Link]

	β	Standard Error	Standardized β coefficients	Partial *r*	*P*	Pearson *r*
**wCST‐LL**	−0.007	0.014	−0.085	−0.079	0.606	−0.004
**Age**	−0.002	0.003	−0.108	−0.100	0.512	−0.247
**Fazekas Score**	−0.060	0.050	−0.193	−0.180	0.236	−0.262
**Baseline score**	−0.376	0.185	−0.274	−0.296*	0.049	−0.295
**Time**	−0.006	0.004	−0.249	−0.250	0.098	−0.327

*p<0.05.

Response to therapy as dependent variable from a multiple regression with predictors’ wCST load, age, Fazekas score, baseline Walk Speed and Time. For each variable, the Βeta, standard error for Βeta and standardized Βeta Coefficient is given together with the significance for this component, the raw Pearson correlation of the dependant variable with response to therapy, and the partial correlation independently of other variables.

**Table 5:** Ordinal regression to predict FAC response to therapy^a^

	OR	95% CI	Wald *χ* ^2^(1)	P value	Pearson *r*
wCST‐LL	0.80	0.66–0.98	4.71	0.030*	−0.155
Age	0.94	0.89–0.98	6.78	0.009*	−0.302*
Fazekas score	1.29	0.64–2.60	0.50	0.478	−0.046
Baseline score	0.21	0.11–0.38	25.54	0.000**	−0.658**
Time	0.94	0.89–1.00	4.37	0.037*	−0.331*

a Response to therapy as dependent variable from an ordinal regression with predictors’ wCST load, age, Fazekas score, baseline Walk speed and time. For each variable, are given the odds ratio (OR), 95% confidence interval for the OR, the χ^2^ together with its significance for this component, and the raw Pearson correlation of the dependant variable with response to therapy.

*Significant *P* < 0.05.

**P < 0.001. The OR for Age implies the odds of recovery decrease by 0.94 for each increase in age of 1 year. Units for Time to Baseline are days, and for wCST cm^3^.

**Table 6:** Multiple regressions to predict MRMI response to therapy (same explanations as Table IV)

	*β*	Standard Error	Standardized *β* coefficients	Partial *r*	*P*	Pearson *r*
wCST‐LL	−0.489	0.209	−0.241	−0.335*	0.024	−0.195
Age	−0.079	0.049	−0.168	−0.241	0.111	−0.125
Fazekas score	0.382	0.768	0.051	−0.076	0.621	0.001
Baseline score	−0.701	0.081	−0.740	−0.798**	0.000	−0.752*
Time	−0.133	0.058	−0.215	−0.330*	0.027	−0.369*

### VLSM

Figure 1 shows the lesion overlap map overlaid on a standard MNI space brain, documenting that the most common lesion site involved the striatocapsular area. Out of the 50 subjects, four lesions had no overlap with any of the other lesions and the maximum number of overlapping lesions was 24. Supporting Information, Figure 2 shows the overlap for voxels lesioned in at least six subjects, that is, the “search volume” for the VLSM analysis. This illustrates that the striatocapsular area, the frontal white matter up to the centrum semiovale, the external capsule, the insular cortex and extensive cortical areas including the precentral gyrus and the frontal opercula were all encompassed in the search volume. A power map from the lesion overlaps with the zero‐thresholded left CST overlaid from the John Hopkins University (JHU) white matter tracts atlas is shown in Supporting Information, Figure 3, illustrating that the CST intersects close to the peak power area.

**Figure 2:**
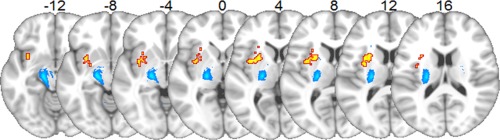
Significant VLSM cluster (yellow) showing lesioned voxels negatively correlated with Walk Speed response to therapy, projected onto the MNI canonical T1‐weighted MRI (see Fig. 1 for details). Only the axial slices with significant voxels are presented (the figure above each slice is the *z* coordinate in mm in MNI space). Statistical significance was determined following permutation correction at *P* < 0.05 FWE correction for multiple comparisons, and controlling for age, Fazekas score, time since stroke onset and baseline Walk speed score as nuisance covariates in the multivariate model (see Methods section). See Table 5 for coordinates, *P* value and anatomical location of the cluster. The canonical JHU cortico‐spinal tract (blue) did not overlap with the cluster. [Color figure can be viewed in the online issue, which is available at http://wileyonlinelibrary.com.]

According to a design that included age, Fazekas scores, time and baseline scores as nuisance covariates, the VLSM analysis revealed a single significant cluster for Walk speed response to therapy, with a *P* value < 0.02. Lesion in this area was significantly correlated with poorer recovery. Table 7 labels the cluster's centre of mass in MNI space, together with the percentage of the cluster labelled according to the Hammers and JHU white matter tracts atlases. The overlaps with the Anatomy are not shown because the cluster did not overlap with any Brodmann area identified in this atlas. Figure 2 depicts this cluster overlaid on a standard MNI template. The areas encompassed included the insula, lateral and anterior putamen and external capsule, and the superior longitudinal, inferior fronto‐occipital and uncinate fasciculi. Of note, the cluster did not overlap with the CST. There were no significant findings with FAC or MRMI.

**Table 7:** Significant cluster from the VLSM analysis of Walk speed response to therapy, obtained from a design including age, Fazekas score, baseline Walk speed and time from stroke to baseline assessment as nuisance covariates (see Methods section)

	Cluster size^a^	Centre of mass^b^	P*	Hammers (anatomy)	JHU (white matter tracts)
Walk Speed	309	[−30,5,4]	<0.02	Insula (67) Putamen (27) Middle frontal gyrus (3)	Inferior fronto‐occipital fasciculus (28) Superior longitudinal fasciculus (24) Uncinate fasciculus (14) Anterior thalamic radiation (3)

Clusters are anatomically labelled by the Hammers and John Hopkins University white matter label tracts (JHU) atlases (rounded % of overlap in brackets). Only overlaps ≥1% are listed

a Cluster size in voxels (corresponds to a volume of 2.47 mL).

b MNI coordinates.

*P value (*P* < 0.05 FWE following uncorrected threshold of *P* < 0.005; see Methods section).

## DISCUSSION

To our knowledge, this is the first study to combine CST damage measurement and VLSM to comprehensively assess the relationship between lesion topography and clinical measures of motricity post‐stroke. To this end, we used the wCST‐LL method [Feng et al., 2015; Zhu et al., 2010] and VLSM [Bates et al., 2003] to assess the role of CST and non‐CST damage, respectively. Because the former assesses the CST in its entire intracranial length rather than locally as the latter does, it is expected to have much greater sensitivity and accuracy to assess the role of CST lesion in behavioural outcome. Our specific goal using this complementary approach was to investigate whether lesion anatomy predicts the response of stroke‐induced impaired walking and mobility to rehabilitation. Another strength of our study is the use of three different outcome measures, namely walk speed as primary outcome and FAC and MRMI, two functional scales assessing distinct daily functions—namely getting out of bed and ambulating and walking under various environments, respectively—as secondary measures. A final strength is the substantial sample size available, affording adequate statistical power.

### General Points

Before discussing the results, it is worth clarifying from the outset that our aim was not to decipher the mechanisms underlying recovery of walking after stroke, but to determine if stroke lesion topography predicts response to gait rehabilitation. We addressed the following pragmatic clinical question: can response to gait therapy be predicted based on stroke lesion topography as determined on standard clinical MRI? Accordingly, we did not assess variables such as functional activation patterns, functional and structural connectivity changes, white matter tract degeneration, or structural changes remote from the lesion such as enlarged cortical thickness or white matter bundles, that all can underlie plastic processes and hence contribute, or impede, functional recovery [Calautti and Baron, 2003; Cramer, 2008; Johansen‐Berg et al., 2002; Sharma et al., 2009]. Although these MRI‐based investigations would be of interest to decipher the mechanisms underlying recovery of walking post‐stroke, they would not contribute to our pragmatic clinical aim.

Three main results emerged from our study. First, baseline scores and time to baseline assessment and less consistently age, predicted response to therapy for some or all clinical variables, confirming these are important covariates to consider when assessing the independent predictive value of lesion topography for behavioural gains. Second, using a multiple regression model accounting for the above covariates plus white matter lesion score, CST damage independently influenced FAC and MRMI response, but not Walk speed. Importantly, of the three clinical measures, Walk speed response was the least well predicted by the five‐variable model, suggesting other variables are operative. Accordingly, assessing non‐CST lesion involvement using VLSM revealed significant findings only for Walk speed, namely a cluster involving the insula, putamen, external capsule and surrounding white matter tracts.

The findings regarding the covariates deserve a brief comment. Baseline score had the strongest influence, consistent with previous studies on post‐stroke walking and ambulation [Burke et al., 2014; Dawes et al., 2008; Jorgensen et al., 1995; Kollen et al., 2005]. That time elapsed since stroke onset also influenced response to therapy was expected given that this study enrolled patients relatively early after stroke, and that recovery slope is steeper at the early post‐stroke stage [Duncan et al., 1992]. Previous studies carried out in the chronic stage also reported an effect of time on recovery [Kollen et al., 2005; O'Shea et al., 2014; Stinear et al., 2007]. Again consistent with previous work [Dobkin et al., 2014; Held et al., 2012; Jorgensen et al., 1995; Lam et al., 2010; Stinear et al., 2007], age impacted—albeit weakly so—recovery of ambulation. Finally, white matter FLAIR hyperintense lesion load did not significantly influence recovery, but was included a priori in the model given its previously reported impact [Held et al., 2012].

### Involvement of the CST

CST damage independently, albeit weakly, predicted FAC and MRMI response to therapy, but not Walk speed. This limited impact of CST damage on gait and ambulation recovery may seem unexpected given the reports regarding the upper limb consistently showing a strong effect [Lindenberg et al., 2012; Nouri and Cramer, 2011; Riley et al., 2011; Stinear et al., 2007]. It is unlikely that our findings are due to inadequate power, since the sample size was similar to that analysed in a previous upper limb impairment study also using wCST‐LL [Zhu et al., 2010], and several‐fold larger than three positive studies of upper limb response to therapy [Lindenberg et al., 2012; Riley et al., 2011; Stinear et al., 2007] that all showed a strongly significant role of the CST. Our findings are in fact entirely consistent with a previous study that reported that CST damage predicted ambulation outcome assessed with FAC [Kim et al., 2013], as well as with all previous reports that assessed the role of CST damage in Walk speed outcome [Jayaram et al., 2012] or response to therapy [Burke et al., 2014; Dawes et al., 2008], which were all negative.

Previous work, also using the wCST‐LL method, found that within the same population of stroke survivors, CST damage was more strongly related to upper limb than lower limb sensorimotor function (assessed with the Fugl‐Meyer scale) (Schlaug et al., unpublished data). The weaker predictive value of CST damage for lower compared to upper extremity outcome might in part reflect the fact that canonical CST templates do not include other descending CSTs such as the cortico‐rubral and the cortico‐tegmental spinal tracts, which have slight differences in their cortical origins compared to the pyramidal tract [Ruber et al., 2012, 2013] and might innervate alpha motoneurons on both sides of the spinal cord, either directly or indirectly, as well as proximal muscles more than distal muscles. As compared to upper extremity sensorimotor function, walking involves quite different motor control processes, including adapted body orientation relative to space and environment, trunk stabilization around the body's centre of mass, generation of alternate leg force to produce a cycling movement, and secure navigation in the surroundings [Perennou and Hillier, 2014], which might account for a limited role of the CST, and an important role for other structures, in this largely automatic function. Conversely, that CST damage influenced FAC and MRMI may reflect the more composite nature of these scales that involve ‘cortical effort’, as they for instance include on top of walking help with the upper limbs and trunk mobility, and are assessed in real, complex environment such as bed surroundings or stairs.

### Involvement of Other Structures

Given the lack of impact of CST damage on Walk speed response, and the limited impact of the clinical variables tested, the finding that a non‐CST located cluster emerged from VLSM is not unexpected. Based on VLSM, involvement of the insula, lateral and anterior putamen and external capsule hindered Walk speed response, independently of the other covariates. Note that the percentages for lesion overlap shown in Table 5 are only approximate because the Hammer's Atlas focuses on grey matter structures and does not consider fine anatomical details. This is particularly true for the “insula” label, which encompasses also the external capsule, claustrum and extreme capsule, leading to gross overestimation of the overlap with the insula proper. Note also that the VLSM cluster did not overlap with the areas of highest statistical power (Supporting Information, Fig. 3), which involve the deep white matter and CST regions, and hence is not the result of intrinsic bias. In addition, the permutation analysis was carried out on the whole search area, not just on the voxels selected by the initial first‐level thresholding, that is, there was no circularity or ‘double‐dipping’ involved [Kriegeskorte et al., 2009]. It is standard in VLSM studies to consider correlations that survive this extremely stringent process as very robust.

The putamen, as a node in the sub‐ cortico‐cortical motor loop, is involved in movement initiation, which is impaired in Parkinson's disease [Alexander et al., 1990; DeLong and Wichmann, 2007], and in the implicit learning and execution of well‐learned sequences (i.e., procedural memory) including walking and balance, accounting for its apparent role in relearning to walk after stroke [Scherder et al., 2011]. It is, therefore, not surprising that damage to the putamen impairs the automatic act of walking. Previously, Alexander et al. [Fig. 2A; 2009] reported an association of gait asymmetry and leg weakness to lesions of the lateral putamen, external capsule and insula in partly recovered stroke patients. Also, changes in putaminal fMRI activations during foot movement were found to correlate well with improvements in walking speed following treadmill‐based rehabilitation after stroke [Enzinger et al., 2009]. Using VLSM, Wu et al. [2015] recently reported that the putamen, insula and external capsule, among other structures, contribute to poor post‐stroke functional outcome, and Cheng et al. [2014] found that lesions to the insula affected global outcome. Overall, therefore, our finding that damage to the putamen, insula and external capsule affects recovery of walking seems consistent with previous work.

The VLSM analysis also suggested that damage to the superior longitudinal, inferior fronto‐occipital and uncinate fasciculi affected Walk speed response. The functions sustained by the two former tracts are not well understood, but they connect the prefrontal and premotor regions to the occipital cortex, and as such could be involved in balance and walking. Accordingly, white matter ischemic lesions particularly involving the frontal lobe are associated with gait impairment [de Laat et al., 2011]. More specifically, damage to the superior longitudinal fasciculus has been linked to decreased postural stability and wide‐based gait in elderly subjects [Scherder et al., 2011]. Regarding the uncinate fasciculus, which connects the anterior part of the frontal lobe to the medial and lateral temporal cortex, its damage in aged people has been linked to decreased step length and walking velocity as well as more generally with apractic gait [Scherder et al., 2011]. Following a stroke, damage to the superior longitudinal and uncinate fasciculi were recently reported to contribute to worse global functional outcome [Wu et al., 2015].

On a physiological standpoint, although the hard‐wired basis for synergistic stepping is engendered in the spinal cord by so‐called ‘central pattern generators’ (CPGs), walking involves a variety of supraspinal areas. Current understanding proposes that supraspinal control may in fact be more important than CPGs for human walking [reviewed by Verma et al., 2012]. Evidence for this interpretation includes the association of gait temporal asymmetry after stroke with posterolateral putamen lesion [Alexander et al., 2009]. More recently, it has also been shown that, together with the pedunculopontine nucleus located in the brainstem, the sub‐thalamic nucleus, which is part of Alexander's motor loop referred to above and as such strongly connected to the putamen and motor cortical areas, plays a significant role in imagined gait in humans [Lau et al., 2015].

### Stroke Side

To ensure optimal statistical power, lesions were flipped so that they all mapped onto the same hemisphere, as is widely done [Cheng et al., 2014; Lo et al., 2010; Zhu et al., 2010]. However, half of the patients had their stroke on either side. Although there is only scant evidence that ambulatory functions are hemisphere‐dependent, this hypothesis cannot be excluded. To address this, we repeated post hoc, the VLSM analysis separately for the left‐ and right‐sided strokes (*n* = 25 in each), which revealed no significant cluster for either (data not shown), likely resulting from the loss of statistical power.

### Mobility, Ambulation and Walk Speed

In this study, we used three different measures of recovery, namely Walk speed as primary outcome, and FAC and MRMI as secondary measures. Although response to therapy for these three scales was significantly inter‐correlated, the correlations between Walk speed and the other two scales were weak, while that between FAC and MRMI was strong but accounted for only ∼50% of the variance, in part, reflecting the fact that these two scales share some items (e.g., walking independently). However, they are constructed to assess different everyday functions, that is, MRMI assesses the ability to move in and get out of bed and walk, and includes items such as turning over in bed, lying to sitting, sitting balance, sitting to standing, standing balance, walking indoors and walking up the stairs, while FAC assesses ambulation in various surroundings including in parallel bars, evaluating the degree of dependency on physical assistance right through to independence. Accordingly, although their relationships with wCST‐LL and several co‐variates are similar (Tables 5 and 6), their relationship with age is different, and differences between MRMI and FAC versus Walk speed (Table 4) are even more striking. On a clinical point of view, it would have no relevance to merge MRMI and FAC into a single compound variable because they are validated and used in whole in daily rehabilitation practice, while Walk speed represents speed of walking in a laboratory setting (i.e., walking forwards on a flat, even floor, in a protected environment). These three scales were prospectively chosen for this study for these specific reasons. Likewise, it would not be clinically acceptable to split these scales into their component items with a view to derive independent dimensions, because they have been prospectively constructed to represent a single overall everyday function (e.g., getting out of bed and ambulate, as at home) and then validated as a single value in extensive investigations.

## LIMITATIONS

Stroke topography varied widely across patients in our sample (Supporting Information, Fig. 2), which could have increased the variance and reduced the statistical strength of this study despite the sizeable sample. However, our sample was gathered prospectively among consecutive referrals to rehabilitation centres, as part of a randomized clinical trial. Accordingly, our sample is representative of routine referrals for post‐stroke gait rehabilitation, making our results clinically relevant. Selecting post hoc a homogeneous sub‐sample based on, for example, stroke topography or etiology would have hindered this clinical relevance and generalizability. Conversely, this variability caused the VLSM search volume to be restricted to a circumscribed zone (Supporting Information, Fig. 2). Of note, the M1 leg area, which is located on the medial surface of the posterior frontal lobe and belongs to the ACA territory, was not part of the search volume, so the influence of its lesion on treatment response could not be assessed. However, consistent with the notion that ACA infarctions are relatively rare, individual analysis of native space MRI showed only two patients with leg area involvement, and this was in fact associated in both cases with extensive sub‐cortical damage (Fig. 1).

Applying VLSM requires that a minimum number of subjects with a lesion in any particular voxel be set a priori, simply because the statistical analysis is based on two‐sample comparisons and the ‘lesioned’ sample has to be reasonably large to make robust inferences. Although there is no strict recommendation on how to determine this threshold in each particular study, we used six subjects in this study because this is a statistically reasonable sample. To further explore this issue, we carried out a post hoc sensitivity analysis using 10 subjects as threshold. The same cluster as with six subjects emerged, although as expected smaller. We also carried out additional post hoc sensitivity analyses using <0.001 as initial default *P* threshold. Again the same cluster emerged, of smaller extent but including the same anatomical structures. The same was also true using a threshold of 10 subjects and *P* < 0.001 as initial default. These sensitivity analyses strongly support the robustness of our VLSM findings. Regarding the topographical accuracy of VLSM, it has been argued that studying patients with large‐artery ischemic strokes might cause several‐mm displacement of the significant clusters because of the intrinsic vascular architecture affecting the shape of resulting infarcts [Mah et al., 2014]. In this situation, distinct approaches to image processing requiring very large datasets and simple, dichotomized outcomes have been proposed [Mah et al., 2014]. However, our study enrolled patients with highly variable stroke mechanisms (including 9 with haemorrhage) and infarct locations and etiologies (including 17 with lacunar infarcts), which would have mitigated any such effect. Minor errors in the precise anatomical localisation of the VLSM cluster cannot however be excluded.

We do not provide formal validation of our findings in two independent samples. Given that the maximum number of patients with a lesion in any given voxel was 24 (Fig. 1), it was not feasible to split the sample in two to assess reproducibility with adequate power. We have, however, carried out an internal validation of the VLSM cluster using the ‘leave‐one‐out’ approach, which showed a stable peak location (centre of mass within 1.4 mm of whole group cluster for 48/50 analyses). Nevertheless, replicability of our findings is an important goal for future studies. Finally, although we identify regions that may help predict treatment response, we do not claim that we can individually classify responders according to lesion location. To be able to do this and for instance report sensitivity and specificity, dichotomized classification of response to therapy as responders and non‐responders would be required. However, there is no universally accepted, validated dichotomized Walk speed, FAC or MRMI response available at this time.

This study was carried out as part of a pragmatic randomised trial assessing whether the benefits of routine physical therapy could be augmented by an ankle–foot orthosis (AFO), custom‐made and fitted by a therapist within a 24‐h period, to provide optimal alignment of the lower limb to the ground during walking [Pomeroy et al., 2012]. Thus both groups received routine physical therapy (treatment‐as‐usual) provided by the clinical physiotherapists. As presented in detail elsewhere [Pomeroy et al., 2016], there were no statistically significant differences between the two groups in the content of physical therapy they received, except that the control group had higher use of off‐the‐shelf AFOs and the experimental group had as expected a higher use of a SWIFT Cast, and there were no statistically significant differences between the groups for any outcome measure, not even evidence of trends (Pomeroy et al. submitted). The experimental device, therefore, did not influence response to therapy, and our findings are, therefore, applicable to standard physical therapy in routine clinical practice.

In our study, there was a wide variance in the nominal number of sessions given to and treatment duration among patients [Pomeroy et al., 2016], which could have influenced response to therapy. However, this was a pragmatic trial conducted in the routine rehabilitation environment where participants were resident either a hospital stroke rehabilitation ward or their own homes. As is commonplace in similar trials, the study could not make it compulsory for therapists to record in a systematic and standardized way each and every minute of routine therapy directed at enhancement of lower limb function they gave to the participants of the trial. Thus, the data collected is for therapy provided when a therapist was present and does not include any additional, self‐administered therapy. Consequently, the data were judged insufficiently accurate to be used as a covariate. Nevertheless, applying standard methods for missing data in the present MRI subset, we found no significant relationship between treatment duration and response to therapy for any of the three clinical measures (data not shown). This is in fact not surprising as there is no clear relationship between the amount of standard post‐stroke physiotherapy and clinical response [English and Veerbeek, 2015], partly because more disabled subjects tend to get more intensive therapy, whilst less affected subjects are trained and instructed to recourse to self‐administered walking and balance exercises. Despite the above caveats, we tested the effects of adding treatment duration as a further covariate in the multivariate analyses, which did not substantially change the results of the wCST‐LL analyses and VLSM.

In our study, the MRI was carried out only after recruitment and start of therapy. This was considered unimportant for the assessment of the predictive value of stroke lesion location for response to therapy because stroke lesions are stable from about 2 weeks after onset (after vasogenic oedema has vanished) up to around 12 weeks (before significant shrinkage occurs) [Gaudinski et al., 2008]. Because of difficulties in obtaining scanning slots for this research study, some scans were carried out slightly later than planned, which could have affected the accuracy of lesion masks in some cases. In the event of real‐life application of our paradigm, however, MRI would need to be carried out before the rehabilitation regimen is decided, in order to guide it. In the clinical setting, if MRI is not available other approaches to predict response of walking speed to gait therapy could be considered such as TMS [Hendricks et al., 2003a, 2003b; Piron et al., 2005], which, however, does not provide information regarding non‐CST located damage.

## CONCLUSION

The findings from this study suggest that strokes affecting the lateral putamen and neighbouring structures reduce response of walking speed to standard rehabilitation, while CST damage has statistically significant, though somewhat limited, impact on two functional scales assessing general mobility and gait.

## Supporting information

Supporting Information Figure 1

Supporting Information Figure 2

Supporting Information Figure 3
