# Supplementary figures and images for: Does stroke location predict walk speed response to gait rehabilitation?
Source: Hum Brain Mapp. 2015 Nov 19;37(2):689–703. doi: 10.1002/hbm.23059 (PMC4738376; doi:10.1002/hbm.23059)

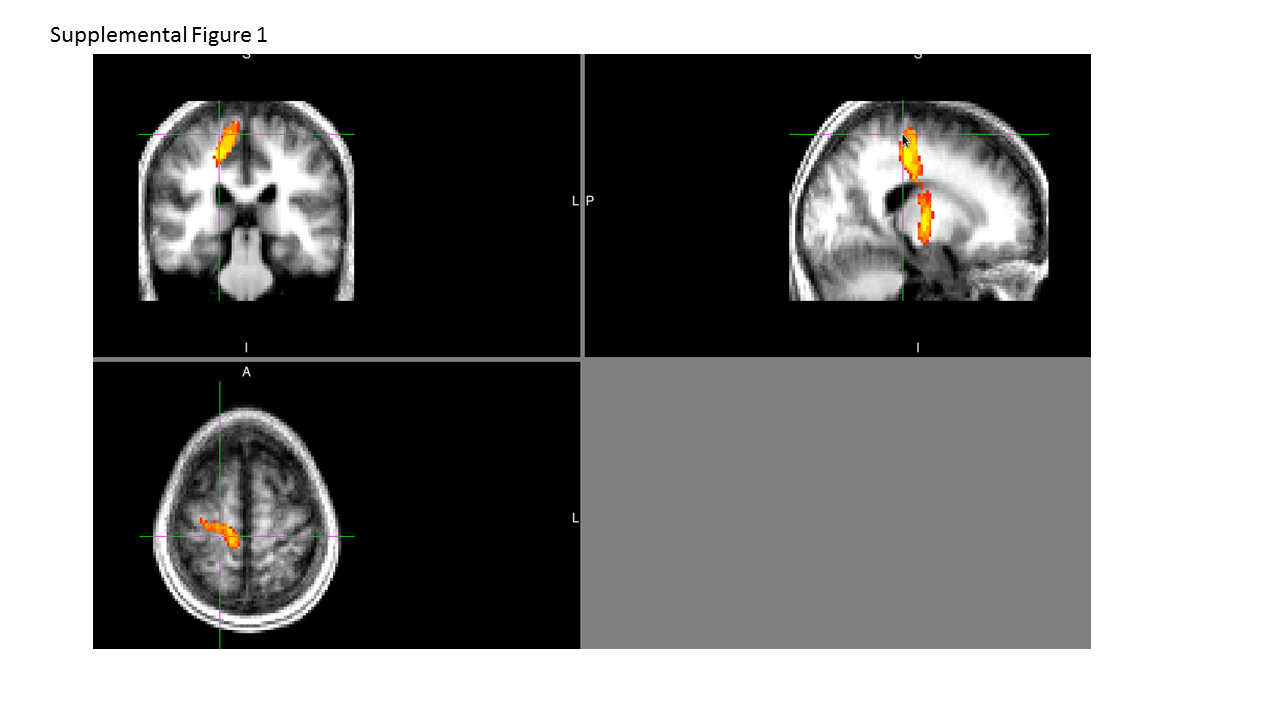

Supplement: Supplementary file 1 — Supporting Information Figure 1 [file HBM-37-689-s001.tif]

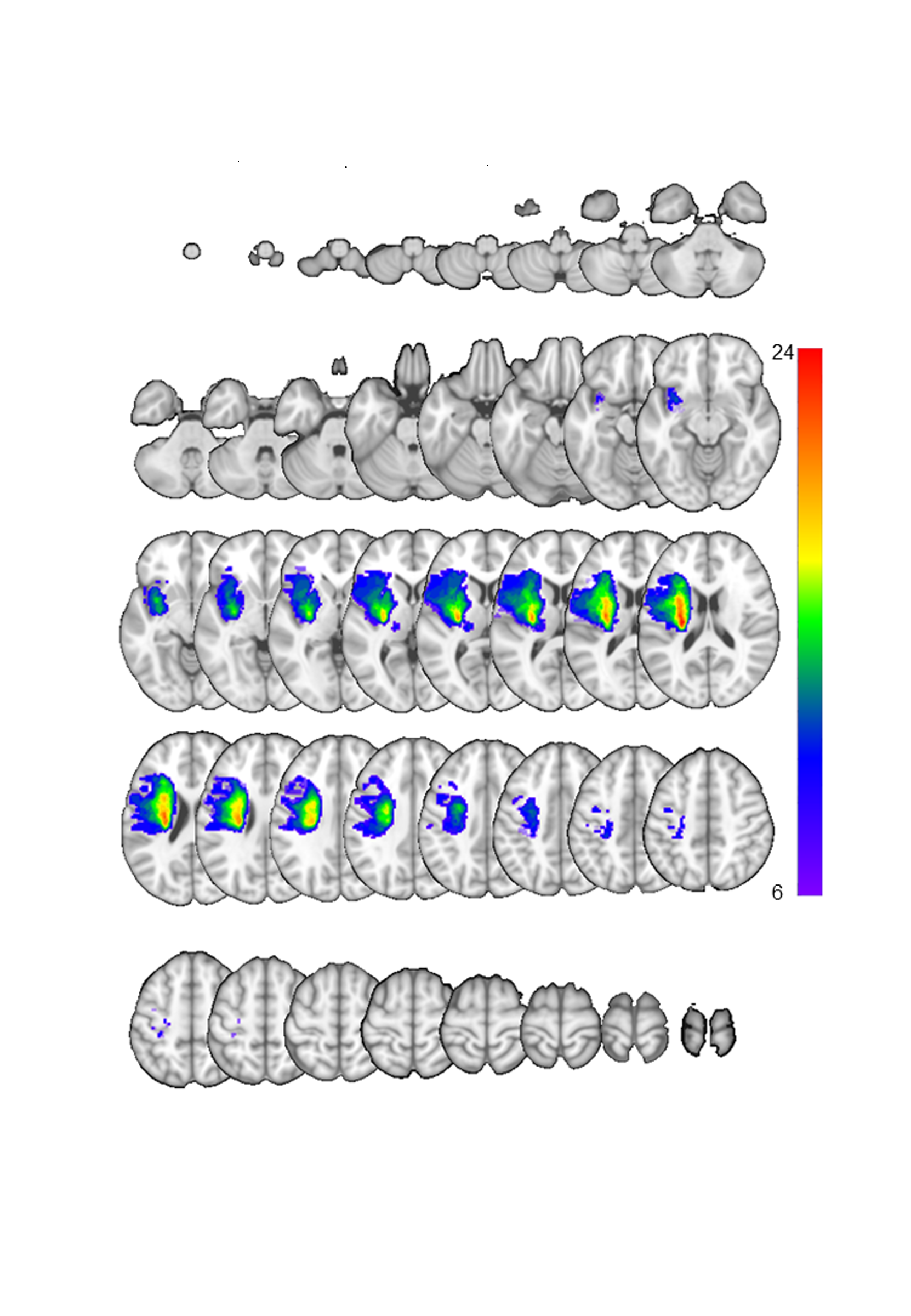

Supplement: Supplementary file 2 — Supporting Information Figure 2 [file HBM-37-689-s002.tif]

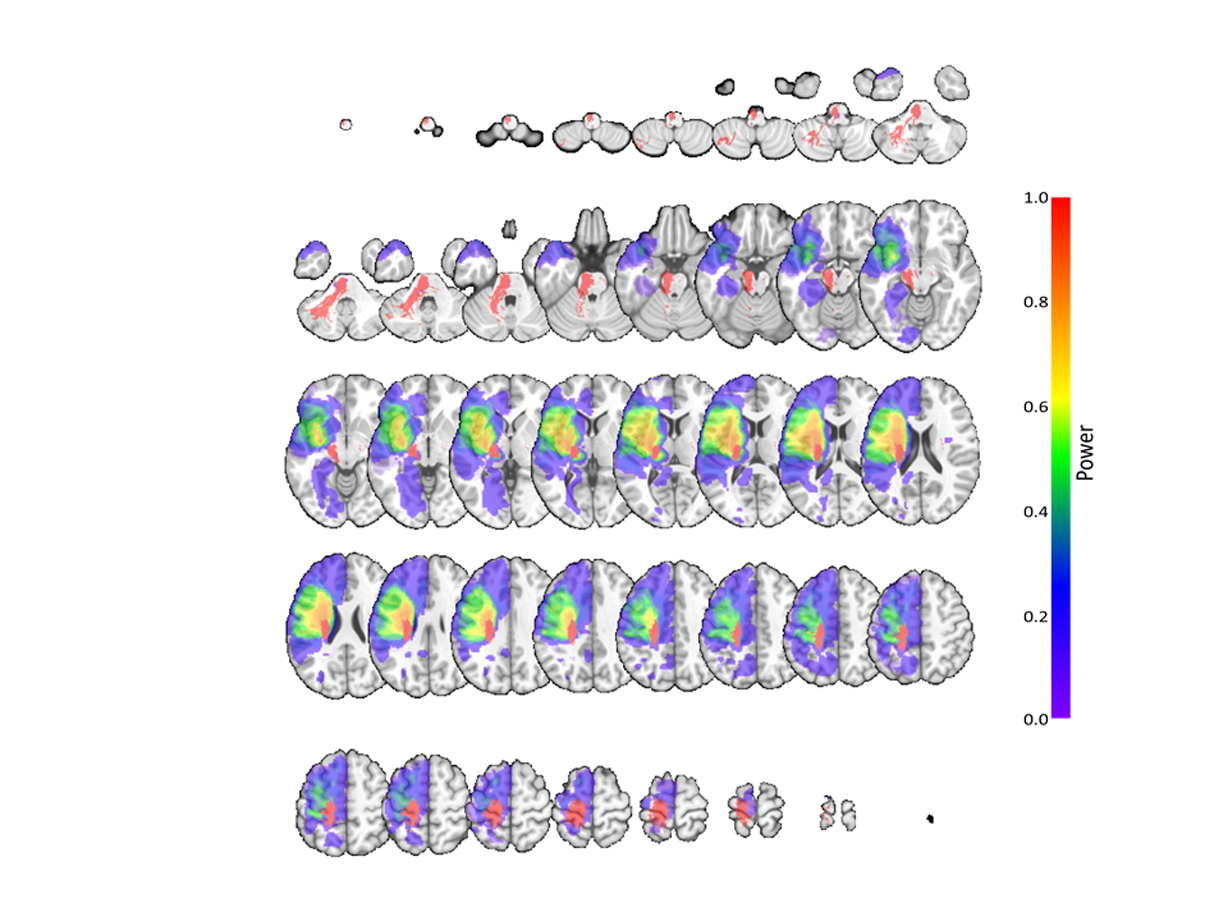

Supplement: Supplementary file 3 — Supporting Information Figure 3 [file HBM-37-689-s003.tif]
